# A Fusion Localization Method based on a Robust Extended Kalman Filter and Track-Quality for Wireless Sensor Networks

**DOI:** 10.3390/s19173638

**Published:** 2019-08-21

**Authors:** Yan Wang, Huihui Jie, Long Cheng

**Affiliations:** Department of Computer and Communication Engineering, Northeastern University, Qinhuangdao 066004, Hebei Province, China

**Keywords:** wireless sensor network, non-line of sight, robust extended Kalman filter, track quality, weighted fusion

## Abstract

As one of the most essential technologies, wireless sensor networks (WSNs) integrate sensor technology, embedded computing technology, and modern network and communication technology, which have become research hotspots in recent years. The localization technique, one of the key techniques for WSN research, determines the application prospects of WSNs to a great extent. The positioning errors of wireless sensor networks are mainly caused by the non-line of sight (NLOS) propagation, occurring in complicated channel environments such as the indoor conditions. Traditional techniques such as the extended Kalman filter (EKF) perform unsatisfactorily in the case of NLOS. In contrast, the robust extended Kalman filter (REKF) acquires accurate position estimates by applying the robust techniques to the EKF in NLOS environments while losing efficiency in LOS. Therefore it is very hard to achieve high performance with a single filter in both LOS and NLOS environments. In this paper, a localization method using a robust extended Kalman filter and track-quality-based (REKF-TQ) fusion algorithm is proposed to mitigate the effect of NLOS errors. Firstly, the EKF and REKF are used in parallel to obtain the location estimates of mobile nodes. After that, we regard the position estimates as observation vectors, which can be implemented to calculate the residuals in the Kalman filter (KF) process. Then two KFs with a new observation vector and equation are used to further filter the estimates, respectively. At last, the acquired position estimates are combined by the fusion algorithm based on the track quality to get the final position vector of mobile node, which will serve as the state vector of both KFs at the next time step. Simulation results illustrate that the TQ-REKF algorithm yields better positioning accuracy than the EKF and REKF in the NLOS environment. Moreover, the proposed algorithm achieves higher accuracy than interacting multiple model algorithm (IMM) with EKF and REKF.

## 1. Introduction

The most popular worldwide positioning system is the Global Positioning System (GPS), but it is not a viable option in some areas, especially in indoor environments, due to the fact that GPS positioning is based on multiple satellites and the positioner cannot acquire accurate signals in indoors environments because of obstructions such as reinforced concrete. GPS technology also relies too much on terminal performance, that is, satellite scanning, acquisition and positioning operations are integrated into the terminal, which results in low positioning sensitivity and high power consumption of the terminal. Meanwhile, wireless sensor networks (WSN) for indoor localization have been drawing more and more attention in recent years. A WSN is an intelligent measurement and control network system composed of a large number of ubiquitous microsensors with communication and computing capabilities. The sensor nodes are intensively deployed in unmanned monitoring areas and can independently complete assigned tasks according to the environment. Because of these excellent characteristics, WSNs have been widely applied to various applications, such as the military field, emergency rescue, environmental monitoring, health care and industrial applications.

As the basic application in WSNs, localization techniques play a key role. Two types of nodes are involved in the positioning process, one are beacon nodes, the other are mobile nodes. Nodes that obtain coordinate information beforehand are called beacon nodes, while the nodes with unknown position coordinates is denoted as mobile nodes. The position of mobile nodes can be estimated by different localization algorithms. According to whether the distance between beacon nodes and mobile nodes is measured during the positioning process, localization algorithms are normally divided into range-based and range-free algorithms. Most of the range-free algorithms are only at the stage of theoretical research and are inadequate to be applied to practice use because they assume uncertainties that cannot be satisfied in reality. The range-based localization algorithms mainly include time difference of arrival (TDOA), time of arrival (TOA), received signal strength (RSS), and angle of arrival (AOA). All the methods mentioned above are widely used in positioning applications.

If the distance or angle measurement between a beacon node and a mobile node is acquired from line-of-sight (LOS) propagation, i.e., the radio propagates along a straight line, the position of the unknown node can be estimated by using conventional algorithms. However, when the direct path between nodes is blocked by obstacles, radio can only reach the receiving terminal after reflection and diffraction, leading to a long propagation path. This phenomenon is known as non-line-of-sight (NLOS) propagation, which occurs especially in indoor environments. When the channel is in NLOS, it will result in a positive bias of the measured distance.

In order to tackle this issue, many NLOS mitigation algorithms are proposed. How to mitigate the NLOS errors is still a difficult task. In this paper, a fusion algorithm is proposed based on robust extended Kalman filter and track quality method. First to make the extended Kalman filter robust, we need to merge the state and the observation equations into a single linear regression model and then utilize maximum likelihood-type estimator. The REKF with an EKF is used in parallel to attain the initial estimates of mobile nodes. The acquired position estimates are considered as observation vectors of the new observation equation in framework of the KF. Then two KFs, both based on the one-step predicted value of the final state estimate at the previous moment, are used for further processing the NLOS error, respectively. Finally, the position estimates are combined based on the fusion algorithm with weighted track quality to obtain the position vector of the mobile node.

The following contributions have been made in this paper:

1) The proposed algorithm fully combines the advantageous features of the two filters to obtain precise localization result. It achieves both efficiency and robustness and even outperforms the EKF in LOS case and REKF in NLOS environment.
2) It only assumes that the measurement noise variance in LOS and the process noise covariance are known in this paper. The prior knowledge of the NLOS errors is not required. Therefore, the proposed algorithm has better latent capacity to reduce localization error.
3) The fusion algorithm heavily exploits the data about the state estimate at previous time, which makes it more immediate and dynamic.
4) An experiment is conducted under indoor environment. The result shows the proposed algorithm performs better than the standard techniques, which indicates the feasibility of the algorithm in the practical environment.

The paper is organized as follows: Section 2 introduces the related work, and Section 3 includes the problem statement and the detailed introduction to the REKF and the fusion algorithm based on the track quality. Then, the proposed algorithm is described in detail in Section 4. The simulation and experimental results are shown in Section 5 and conclusions are drawn in Section 6.

## 2. Related Works

A traditional method of positioning estimation is the EKF [1], which expands the nonlinear model in Taylor series near the state estimates and truncates it in the first order to realize the linearization of the model. Standard EKF based on the above linear model achieves high accuracy in LOS environment. However, when the channel is in NLOS, the EKF algorithm displays large localization errors due to the deviation of measurement data. Many NLOS mitigation methods [2,3,4,5,6,7,8,9,10,11] have been proposed for the location of the mobile nodes. In [2], the unscented transformation is applied to the standard Kalman filter system to generate the unscented Kalman filter (UKF), which achieves great estimation performance. An adaptive iterated unscented Kalman filter (AIUKF) is presented in [3] for target positioning, which improves the performance by combining iterative strategy and adaptive factor. In [4], the particle filter (PF) based on the Monte Carlo method [5,6,7] is used for positioning. It exploits the information of a group of random samples to approximate the probability density function of the state. The particle filter achieves good positioning performance in non-Gaussian system while requiring a large number of samples. In [8,9], all the range measurements are grouped and the final state estimation is obtained based on the fusion of location estimates of these subgroups. In contrast, the authors in [10] consider detecting and discarding the range measurement from the beacon nodes in NLOS condition. Reference [11] also combines the advantages of these two methods, meaning the NLOS error detection technology is used for different subgroups so that the accepted data can be weighted with different criterions. However, most of the above mentioned approaches achieve precise performance only in a specific noise distribution environment, which is not realistic.

Some algorithms [12,13,14,15] that rely on little knowledge of pdf of the NLOS error are used for accurate positioning. Reference [12] proposes a bisection-procedure-based algorithm to acquire exact localization results, which neither require statistics about the NLOS bias nor any prior knowledge about LOS and NLOS links. Considering the limitation of the convex optimization applied in TDOA systems for NLOS error mitigation [16,17,18], the authors in [13] present a novel method to transform the TDOA model into the TOA model and such that a new constrained semi-definite programming method is used to effectively mitigate the NLOS error in TDOA system. It achieves a high positioning accuracy while requiring little prior information about the NLOS error. In [14] a low-complexity algorithm based on Sparse Pseudo-input Gaussian Process (SPGP) is proposed to directly mitigate the bias of NLOS without information about the environment. Reference [15] employs both the angle of arrival (AOA) and phase difference of arrival (PDOA) information to acquire the localization result. It establishes virtual stations to convert the NLOS paths into LOS paths such that achieves better performance than some traditional positioning algorithms.

Many approaches using multiple filters in parallel are effective to deal with the effect of different types of noise, among which the interacting multiple model (IMM) [19] is extensively used in localization methods such as the Global Positioning System [20]. The IMM algorithm assumes that the target has multiple motion states and each motion state corresponds to a model. The state of the target at any time can be represented by one of the given models. The transformation of motion states and the switching of motion models are determined by Markov probability transfer matrix. The final result is obtained by weighted fusion of filtering results of multiple models based on the probability of each model which can be adjusted adaptively in the filtering process, thus achieving more accurate positioning. In [19], a localization method with NLOS mitigation is proposed using the KF-based IMM algorithm. It includes LOS and NLOS models to estimate the state with two parallel Kalman filters. Reference [21] exploits the data fusion algorithm and extended Kalman-based IMM to acquire more accurate location estimation in the LOS and NLOS case. Reference [22] presents an IMM algorithm reweighted by an Expectation-Maximization algorithm (EM), which is an optimization algorithm based on maximum likelihood estimation theory. References [23,24] combine particle filtering with IMM. Each model in IMM uses a standard particle filter where the number of particles is fixed. The new method can deal with non-Gaussian noise and implement good performance in NLOS environment.

## 3. Problem Statement

### 3.1. Signal Model

In this section, some technical details will be introduced. Assume that M beacon nodes are randomly distributed in the field to detect the range signal from the mobile node. The coordinate of *m-th* beacon nodes is $(x_{m},y_{m}),m=1,\ldots ,M$. The mobile node is moving randomly on a 2D-plane with state vector $x(k)={\left[x(k)\;y(k)\;\dot{x}(k)\;\dot{y}(k)\right]}^{T}$ at each time step *k, k* = *1,…,K,* where (x(k),y(k)) denote the position and $\left(\dot{x}(k),\dot{y}(k)\right)$ the velocity of the mobile node. Its movement is described by the change of x(k) at time step *k* according to [25]:

(1) x(k)=Ax(k−1)+Gw(k−1)

where, $A=\left[\begin{array}{cc} I_{2} & \Delta t\cdot I_{2}\\ 0 & I_{2} \end{array}\right],G=\left[\begin{array}{c} \Delta t^{2}/2\cdot I_{2}\\ \Delta t\cdot I_{2} \end{array}\right]$, Δt is the sampling period and $I_{N}$ is the N×N identity matrix. The process noise w(k−1) is assumed zero-mean, white Gaussian with covariance matrix $Q(k)=\sigma_{w}^{2}I_{2},k=1,\ldots ,K$. The transition matrix A describes the movement of mobile node between the adjacent moment.

The true Euclidean distance $d_{m}(k)$ between the mobile node and the beacon node at time *k* can be expressed as:

(2) $$d_{m}(k)=\sqrt{{\left(x_{m}-x(k)\right)}^{2}+{\left(y_{m}-y(k)\right)}^{2}}$$

Then, in LOS condition, the range measurement corresponding to time of flight (TOF) data can be modeled as:

(3) $$d_{m}^{LOS}(k)=d_{m}(k)+n_{LOS}$$

where $n_{LOS}$ is the measurement noise modeled as a white Gaussian noise with zero mean and variance $\sigma_{LOS}^{2}$, i.e., $N(0,\sigma_{LOS}^{2})$.

The probability density function (PDF) of $n_{LOS}$ usually can be expressed as follows:

(4) $$f(\tau_{LOS})=\frac{1}{\sqrt{2\pi \sigma_{LOS}^{2}}}\exp(-\frac{\tau_{LOS}^{2}}{2\sigma_{LOS}^{2}})$$

When the obstacles prevent the signals from arriving at the beacon nodes, NLOS propagation occurs, leading to bigger distance value than the true. Thus, the distance measurement of the *m-th* beacon node at time *k* is modeled as:

(5) $$d_{m}^{NLOS}(k)=d_{m}(k)+n_{LOS}+n_{NLOS}$$

In a NLOS propagation environment, the error of NLOS $n_{NLOS}$ generally obeys a Gaussian distribution with positive mean value, a uniform distribution or an exponential distribution. The specific models are shown below.

The PDF of $\tau_{NLOS}^{norm}$ which obeys the Gauss distribution $\left(\tau_{NLOS}^{norm}∼N(\mu_{NLOS},\sigma_{NLOS}^{2})\right)$ is given by:

(6) $$f(\tau_{NLOS}^{norm})=\frac{1}{\sqrt{2\pi \sigma_{NLOS}^{2}}}\exp(-\frac{{\left(\tau_{NLOS}^{norm}-\mu_{NLOS}\right)}^{2}}{2\sigma_{NLOS}^{2}})$$

The PDF of $\tau_{NLOS}^{unif}$ which obeys the uniform distribution $\left(\tau_{NLOS}^{unif}∼U(u_{\min},u_{\max})\right)$ is given by:

(7) $$f(\tau_{NLOS}^{unif})=\left\{\begin{array}{l} \frac{1}{u_{\max}-u_{\min}},\;u_{\min}\leq \tau_{NLOS}^{unif}\leq u_{\max}\\ \;\;0,\;\;\;\;\;\;else \end{array}\right\}$$

The PDF of $\tau_{NLOS}^{\exp}$ which obeys the exponential distribution $\left(\tau_{NLOS}^{\exp}∼E(\lambda )\right)$ is given by:

(8) $$f(\tau_{NLOS}^{\exp})=\left\{\begin{array}{l} \frac{1}{\lambda }e^{-\frac{\tau_{NLOS}^{\exp}}{\lambda }},\;\tau_{NLOS}^{\exp}\geq 0\\ 0,\;\;\;\;\;\;\;\;\tau_{NLOS}^{\exp}\leq 0 \end{array}\right\}$$

### 3.2. Introduction of REKF

The robust extended Kalman filter (REKF) [26,27] combines EKF with robust prediction technology to make EKF robust. By applying the robust technology to the NLOS problem, the REKF can improve performance with respect to EKF in NLOS environment. Its functionality can be briefly described as follows: firstly, we rewrite the nonlinear EKF equations into a linear regression model, which in general is easier to tackle and may acquire near-optimal filtering performance. Then, robust regression techniques that are suitable for handling corrupt data to solve for the parameter of interest, i.e., the unknown state vector, are employed. Some known robust regression techniques [28,29,30,31,32] can be exploited to increase the robustness of the algorithm. Particularly, this paper consider the approach that applying Huber’s robust M-estimator [31,32] (maximum likelihood-type estimator) to robustify the EKF, which limits the effect of outliers. The method can obtain the parameter of interest by solving the derivative of the negative log-likelihood function of a so-called nonlinear score function for zero.

The expression of the score function and the selection of the parameter in it are the key performance factors. One of the most popular score function choices is the soft-limiter [33] that is linear in an interval starting from zero and remains constant after exceeding a certain threshold. This constant value is called clipping point which has to be chosen appropriately. However, this kind of score function minimizes the maximum asymptotic variance of the NLOS error distribution and leads to a biased estimate when the NLOS error distribution has a non-zero mean. An alternative function called “re-descending” score function is derived in [34] for asymmetric noise distribution. It reaches zero beyond a second threshold. The M-estimator based on this score function can have an unbiased estimate by tuning of the clipping points appropriately. For the fact that the range measurements always have a positive bias due to NLOS propagation, this paper considers the approach based on the re-descending score function. Selection of clipping points can adjust the effectiveness and robustness of the estimator in LOS/NLOS case. Details can be found in [31,32]. It is worth noting that robustness in NLOS and the efficiency in LOS are contradictory goals which need a trade-off according to the actual requirements. The specific filtering process of REKF is discussed later in Section 4.

### 3.3. Introduction of Fusion Algorithm Based on the Track Quality

The REKF has robustness in NLOS propagation while the EKF has efficiency in LOS propagation. Therefore, this paper addresses a fusion algorithm based on track quality to combine these two algorithms to obtain more accurate localization result. To describe the track quality, first we define a variable called the normalized distance function, whose value represents the quality of the track. It is to be noted that the smaller value corresponds to the better track quality because the function is acquired based on the innovation of state prediction and its covariance. At every moment, the fusion algorithm feeds back the one-step predicted value of the fusion state estimation at the previous moment to the local filter. Thus, the local filter can calculate the normalized distance function based on the feedback information. The fusion center assigns weights through the evaluation of tracking quality of different filters to realize weighted fusion. Because of the real-time and dynamic nature of the algorithm, more accurate coordinates of the mobile node can be obtained.

## 4. Proposed Algorithm

**Figure 1:**
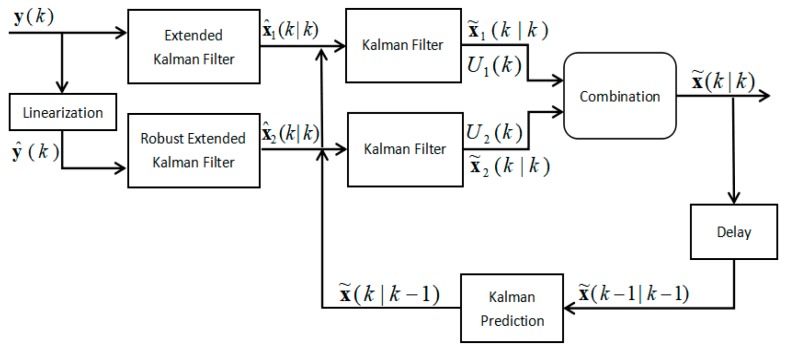
The flowchart for the proposed algorithm.

As shown in Figure 1, the input of the algorithm is the distance measurement y(k) at time step *k* while the output is position vector $\tilde{x}(k|k)$. Firstly, EKF and REKF are used in parallel to process the measurements in the observations. They have the same input, but the difference is that REKF needs to rewrite the non-linear EKF equation into a linear regression model, so as to facilitate the use of robust techniques. After preliminary filtering of measurements, two coordinate estimates are derived from EKF and REKF, respectively. Consider the two location estimates as measurements so acquire the new measurement equation in the filtering process. Then two KFs can be used to filter the two position estimates separately based on the feedback information of final state estimate of previous time step. The position estimates of KFs are combined using the fusion algorithm based on the track equality to yield the final position coordinate of the mobile node.

### 4.1. General Concept

Let y(k) denotes the vector of distance measurement, then the measurement equation can be expressed as:

(9) y(k)=h(x(k))+v(k),k=1,2,…,K

where $h(x(k))={\left[d_{1}(k),d_{2}(k),\ldots ,d_{M}(k)\right]}^{T}$ and $d_{m}(k)$ is given in Equation (2). The noise vector v(k) includes the sensor noise and the perturbations due to NLOS propagation. The measurement covariance matrix R(k) is defined as:

(10) $$R(k)=diag[\sigma_{1}^{2},\sigma_{2}^{2},\ldots ,\sigma_{M}^{2}]$$

where the $\sigma_{m}^{2}$ in R(k) is modeled as:

(11) $$\sigma_{m}^{2}=\{\begin{array}{c} \;\sigma_{G}^{2},\;\;m\text{-}th\;node\;is\;in\;LOS\\ \sigma_{G}^{2}+\sigma_{\eta }^{2},\;m\text{-}th\;node\;is\;in\;NLOS \end{array}$$

The $\sigma_{G}^{2}$ and $\sigma_{\eta }^{2}$ refer to the sensor noise variance and NLOS noise variance, respectively. Assumption that the $\sigma_{G}^{2}$ is known and the $\sigma_{\eta }^{2}$ is unknown is accepted in this paper.

### 4.2. Extended Kalman Filter (EKF)

The EKF steps are divided into two phases: the prediction phase and the update phase.

Step 1: Kalman Prediction

In this stage, a prior estimate of the next time is derived from the current state and error covariance:

(12) $${\hat{x}}_{1}(k|k-1)=A{\hat{x}}_{1}(k-1|k-1)$$

(13) $$P_{1}(k|k-1)=AP_{1}(k-1|k-1)A^{T}+GQ(k)G^{T}$$

(14) $$H_{1}(k)={\frac{\partial h(x(k))}{\partial x(k)}|}_{x(k)={\hat{x}}_{1}(k|k-1)}$$

(15) $$v_{1}(k)=y(k)-h({\hat{x}}_{1}(k|k-1))$$

(16) $$S_{1}(k)=H_{1}(k)P_{1}(k|k-1)H_{1}^{T}(k)+R_{1}(k)$$

Step 2: Kalman Update

The updating step is computed to obtain the posterior state estimation ${\hat{x}}_{1}(k|k)$ and $P_{1}(k|k)$ with the prior state estimation:

(17) $$K_{1}(k)=P_{1}(k|k-1)H_{1}^{T}(k)S_{1}^{-1}(k)$$

(18) $${\hat{x}}_{1}(k|k)={\hat{x}}_{1}(k|k-1)+K_{1}(k)v_{1}(k)$$

(19) $$P_{1}(k|k)=(I_{4}-K_{1}(k)H_{1}(k))P_{1}(k|k-1)$$

In practice, the covariance Q(k) and R(k) are unknown and need to be set beforehand based on the empirical information.

### 4.3. Robust Extended Kalman Filter (REKF)

Step 1: Kalman Prediction

The prediction steps of REKF is similar to the EKF. They are run in parallel:

(20) $${\hat{x}}_{2}(k|k-1)=A{\hat{x}}_{2}(k-1|k-1)$$

(21) $$P_{2}(k|k-1)=AP_{2}(k-1|k-1)A^{T}+GQ(k)G^{T}$$

(22) $$H_{2}(k)={\frac{\partial h(x(k))}{\partial x(k)}|}_{x(k)={\hat{x}}_{2}(k|k-1)}$$

(23) $$v_{2}(k)=y(k)-h({\hat{x}}_{2}(k|k-1))$$

(24) $$S_{2}(k)=H_{2}(k)P_{2}(k|k-1)H_{2}^{T}(k)+R_{2}(k)$$

Since the $\sigma_{\eta }^{2}$ is unknown in practice, this paper proposes replacing it by a multiplicative factor of $\sigma_{G}^{2}$ such that REKF with bigger value of the measurement covariance matrix $R_{2}(k)$ can cope with the large NLOS outliers.

Step 2: Rewrite EKF Equation

Whenever possible, converting the nonlinear model into a linear one allows us to apply the robust techniques to the filter. To do so, rewrite the state and observation Equations (1) and (9) as:

(25) $$\left[\begin{array}{c} I_{4}\\ H_{2}(k) \end{array}\right]x(k)=\left[\begin{array}{c} A{\hat{x}}_{2}(k-1|k-1)\\ y(k)-h({\hat{x}}_{2}(k|k-1)+H_{2}(k){\hat{x}}_{2}(k|k-1)) \end{array}\right]+b(k)$$

where $b(k)=\left[\begin{array}{c} A(x(k-1)-{\hat{x}}_{2}(k-1|k-1))+Gw(k-1)\\ -v(k) \end{array}\right]$ such that the covariance matrix of b(k) is:

(26) $$\begin{array}{ll} E\left[b(k)b^{T}(k)\right] & =\left[\begin{array}{cc} P_{2}(k|k-1) & 0\\ 0 & R_{2}(k) \end{array}\right]\\ & =C(k)C^{T}(k) \end{array}$$

where the C(k) can be obtained by using the Cholesky decomposition. Then multiplying Equation (25) with $C^{-1}(k)$ generates the linear regression model:

(27) $$\hat{y}(k)=S(k)x(k)+\hat{u}(k)$$

where:

(28) $$\begin{array}{l} \hat{y}=C^{-1}(k)\left[\begin{array}{c} {\hat{x}}_{2}(k|k-1)\\ y(k)-h({\hat{x}}_{2}(k|k-1)+H_{2}(k){\hat{x}}_{2}(k|k-1)) \end{array}\right]\\ \;\;\;\;S(k)=C^{-1}(k)\left[\begin{array}{c} I_{4}\\ H_{2}(k) \end{array}\right],\;\hat{u}(k)=-C^{-1}(k)b(k) \end{array}$$

Applying the least-squares to the model, we can get the solution of the equation as follows, which is equivalent to EKF filtering:

(29) $${\hat{x}}_{2}(k|k)={\left(S^{T}(k)S(k)\right)}^{-1}S^{T}(k)\hat{y}(k)$$

Step 3: Robust Regression Algorithm

In this step, the time index *k* is omitted for simplicity. At each time step, if the PDF of $\hat{u}$, i.e., $f_{V}(\hat{u})$, is known, the estimate for x can be obtained by maximum likelihood estimate (MLE). And the estimate ${\hat{x}}_{MLE}$ is the solution of:

(30) $$\sum_{i=1}^{\dim(x)+M}{\left[S\right]}_{ij}\varphi ({\hat{u}}_{i})=0,j=1,\ldots ,\dim(x)$$

where ${\left[\cdot \right]}_{r}$ denotes the *r-th* component of a vector, dim(x) is the dimension of vector x, ${\hat{u}}_{i}=\hat{y}-\sum_{j=1}^{\dim(x)}{\left[S\right]}_{ij}x_{j},i=1,\ldots ,\dim(x)+M$ and $\varphi ({\hat{u}}_{i})=-\frac{{f^{\prime }}_{V}({\hat{u}}_{i})}{f_{V}({\hat{u}}_{i})}$ is the location score function. However, in practice the prior statistic of $f_{V}({\hat{u}}_{i})$ is poor especially in indoor environment. Moreover, the residuals of measurements are linearly weighted as show in Equation (30), achieving poor performance in NLOS condition. Thus, this paper proposes to replace the location score function with a robust one called redescending score function [34,35], given as:

(31) $$\psi (u_{i})=\left\{ \begin{array}{l} u_{i},\;\;\;\;\;\;\;\;\;\;\;\;\;\left|u_{i}\right|\leq c_{1}\\ b\tanh[0.5b(c_{2}-\left|v\right|)]\mathrm{sgn}(u_{i}),\;c_{1}<\left|u_{i}\right|\leq c_{2}\\ 0,\;\;\;\;\;\;\;\;\;\;\;\;\;\;\left|u_{i}\right|\geq c_{2} \end{array}\right.$$

where *b* is chosen to ensure continuity of function ψ. $c_{1}$ and $c_{2}$ are the clipping points of the score function, whose values can map the intensity of robustness. Note that the effectiveness in nominal case (LOS) and robustness in NLOS environment are contradictory indicators. It always requires a trade-off in the algorithm. Since the proposed algorithm combines the REKF with a EKF, the selection of parameters is based on the principle of enhanced robustness. Hence, the clipping points of redescending score function are set to $c_{1}=0.6$ and $c_{2}=0.8$ while b=−2.474. Then compute the state estimate by iterative Newton-Raphson steps based on the proposed score function. The process of the algorithm is discussed as follows:

At Step 1, obtain the initial state estimate, the index l represents the number of iterations:

(32) $${\hat{x}}_{l}={\left(S^{T}S\right)}^{-1}S^{T}\hat{y},l=0$$

At Step 2, compute the noise residuals $\tilde{\hat{u}}$, i.e.,:

(33) $$\tilde{\hat{u}}=\hat{y}-S{\hat{x}}_{l}$$

At Step 3, the scale of the noise is estimated, given as:

(34) $${\hat{\sigma }}_{U}=1.48mad(\tilde{\hat{u}})$$

where $mad(\tilde{\hat{u}})=E\left(\left|\tilde{\hat{u}}-E(\tilde{\hat{u}})\right|\right)$ is the mean absolute deviation. The data in REKF can be normalized by the scale estimate such that they are adapt to the redescending score function.

At Step 4, the state estimate is updated by the Newton-Raphson method, which is based on the score function evaluated at the normalized residuals:

(35) $${\hat{x}}_{l+1}={\hat{x}}_{l}+\mu {\left(S^{T}S\right)}^{-1}S^{T}\psi (\tilde{\hat{u}}/{\hat{\sigma }}_{U})$$

At step 5, calculate the norm of the previous and actual position estimate as follows:

(36) $$\left\|{\hat{x}}_{l+1}-{\hat{x}}_{l}\right\|=\sqrt{\sum_{j=1}^{\dim({\hat{x}}_{l+1}-{\hat{x}}_{l})}{\left({\left[{\hat{x}}_{l+1}-{\hat{x}}_{l}\right]}_{j}\right)}^{2}}$$

Stop the algorithm when the norm is smaller than the required precision and output the estimate. Otherwise, repeat steps 2–5 until the convergence is achieved.

### 4.4. Kalman Filter with New Measurement Equation

In this step, we consider the filtering results as two relatively accurate measurements (i.e., the measurements are obtained from the situation where most or all of the nodes are in LOS, which may not be the case in practice.), and further process the estimation error in the KF framework.

To do this, the new measurement equation at time step *k* is expressed as:

(37) $$z_{p}(k)=B\tilde{x}(k)+\xi_{p}(k),\;\;p=1,2\;\;\;$$

where $z_{p}(k)\in [{\hat{x}}_{1}(k)\;for\;p=1,{\hat{x}}_{2}(k)\;for\;p=2]$, $B=\left[\begin{array}{cccc} 1 & 0 & 0 & 0\\ 0 & 1 & 0 & 0\\ 0 & 0 & 1 & 0\\ 0 & 0 & 0 & 1 \end{array}\right]$. The noise error $\xi_{p}(k)$ is complicated and there is no need to know much information about it in the algorithm. In the paper just approximate the innovation covariance matrix as:

(38) $$\tilde{S}(k)=BP(k|k-1)B^{T}+\sigma_{G}^{2}I_{4}$$

The prediction step of filtering process is the same as conventional KF, given as:

(39) $$\tilde{x}(k|k-1)=A\tilde{x}(k-1|k-1)$$

(40) $$\tilde{P}(k|k-1)=A\tilde{P}(k|k-1)A^{T}+GQ(k)G^{T}$$

(41) $$\tilde{z}(k|k-1)=\tilde{B}\tilde{x}(k|k-1)$$

(42) $$e_{p}(k)=z_{p}(k)-\tilde{z}(k|k-1),\;p=1,2$$

(43) $$\tilde{S}(k)=BP(k|k-1)B^{T}+\sigma_{G}^{2}I_{4}$$

where $e_{p}(k)$ is the innovation of p-th KF and the innovation covariance matrix are both estimated by $\tilde{S}(k)$. The update step is performed in the next subsection.

### 4.5. Combination Based on Track Quality

Firstly, at time step *k* define the normalized distance function as:

(44) $$d_{p}(k)=e_{p}^{T}(k){\tilde{S}}^{-1}(k)e_{p}(k),\;p=1,2$$

It is used to describe the track quality, which is expressed as:

(45) $$U_{p}(k)=\alpha U_{p}(k-1)+(1-\alpha )d_{p}(k),\;p=1,2$$

where α is the historical weight factor with a value of 0 to 1. Its value has little effect on the position estimation of the algorithm, which will be verified in the simulation process.

At each time step either of the EKF and REKF which obtains precise state estimate will lead to smaller innovation and higher track quality. Then it should be overweighted to acquire the final result. Here the weights are assigned as follows:

(46) Wp(k)=2^{−Up(k)}, p=1,2

To get the state estimate, the weights need to be normalized:

(47) $$W^{p}(k)=\frac{W^{p}(k)}{\sum_{\hat{p}=1}^{2}W^{\hat{p}}(k)},\;p=1,2$$

Then the update step can be carried out.

The Kalman gain is obtained by:

(48) $$\tilde{K}(k)=\tilde{P}(k|k-1)B^{T}{\tilde{S}}^{-1}(k)$$

The posterior covariance matrix is calculated as:

(49) $$\tilde{P}(k|k)=(I_{4}-K(k)B)\tilde{P}(k|k-1)$$

The posterior state estimates are updated as follows:

(50) $${\tilde{x}}_{1}(k|k)=\tilde{x}(k|k-1)+\tilde{K}(k)e_{1}(k)$$

(51) $${\tilde{x}}_{2}(k|k)=\tilde{x}(k|k-1)+\tilde{K}(k)e_{2}(k)$$

The final position estimate after weighted fusion can be obtained as:

(52) $$\tilde{x}(k|k)=W^{1}(k){\tilde{x}}_{1}(k|k)+W^{2}(k){\tilde{x}}_{2}(k|k)$$

## 5. Simulation and Experimental Results

### 5.1. Simulation Results

In this section, simulation results are provided to demonstrate the validity of the proposed algorithm. Beacon nodes are randomly deployed in the 100 × 100 $m^{2}$ area where a mobile node moves in a curved trajectory. The simulation platform is MATLAB, and the propagation of NLOS errors between beacon nodes and the mobile node is randomly generated with the probability of LOS occurrence ε. The EKF algorithm, REKF algorithm and IMM algorithm using the EKF with an REKF in parallel are computed for comparison purposes. This paper considers the Root Mean Square Error (RMSE) obtained from 1000 Monte Carlo runs as a performance metric, which is expressed as:

(53) $$RMSE=\sqrt{\frac{1}{T_{n}K}\sum_{i=1}^{T_{n}}\sum_{k=1}^{K}\left({\left({\tilde{x}}_{i}(k)-x(k)\right)}^{2}+{\left({\tilde{y}}_{i}(k)-y(k)\right)}^{2}\right)}$$

where $T_{n}=1000$, K=1000, [x(k),y(k)] is the true position of the mobile node at time step *k* and $\left[{\tilde{x}}_{i}(k),{\tilde{y}}_{i}(k)\right]$ is the position estimate at time step *k* for the *i-th* Monte Carlo run.

In the following section, the simulation experiments are conducted under different environment, i.e., the NLOS errors obey various distribution and different values of the parameters in the algorithm are chosen in the simulation.

#### 5.1.1. The Effect of Historical Weight Factor

**Figure 2:**
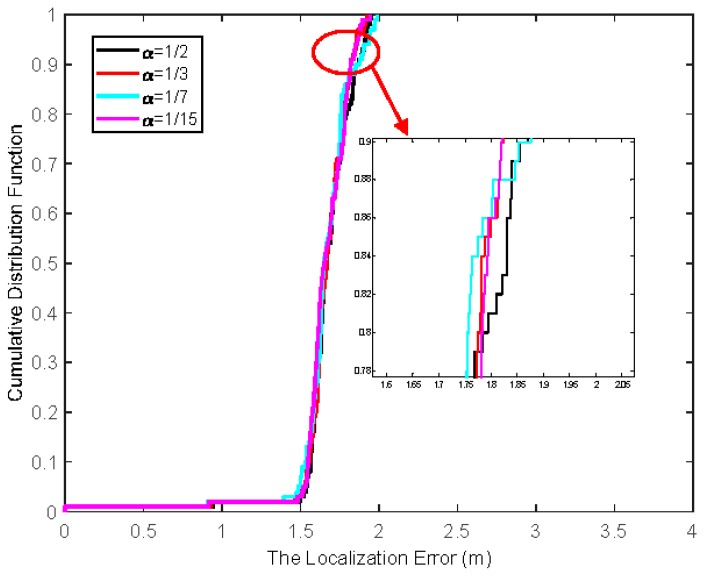
CDF of localization errors for different value of α.

The first step is to investigate the impact of historical weight factor α on the localization error. When the number of beacon nodes is seven and the NLOS error obeys a Gaussian pdf with mean $\mu_{\eta }=3m$ and standard deviation $\sigma_{\eta }=4m$, the cumulative distribution function (CDF) of the localization error is depicted in Figure 2. It can be seen that the algorithm is not sensitive to the value of the historical weight factor. Consider α=1/3 in the following simulation.

#### 5.1.2. The NLOS Errors Obey Gaussian Distribution

**Figure 3:**
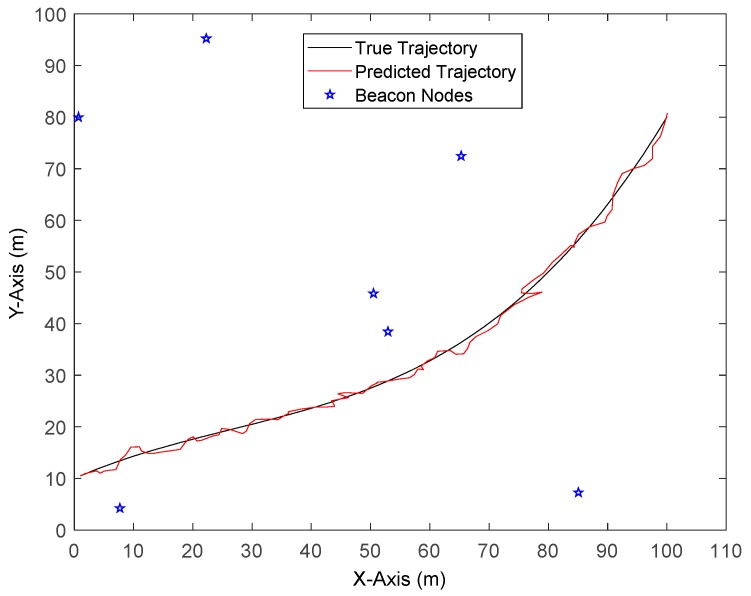
The localization result of REKF-TQ.

The default parameters in the simulation of NLOS errors obeying a Gaussian Distribution are shown in Table 1, Figure 3 displays the localization results by a one-time run of REKF-TQ. It can be seen that the proposed algorithm achieves accurate trajectory tracking while the beacon nodes are randomly distributed in the area.

**Figure 4:**
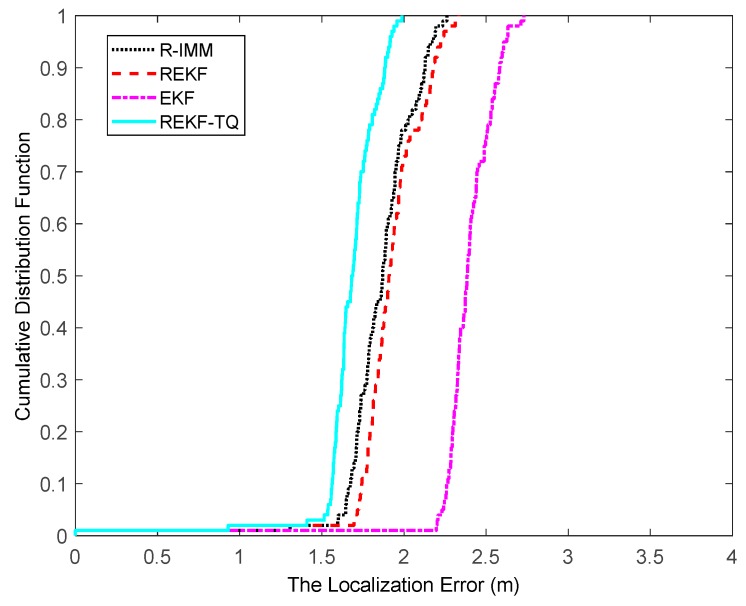
The localization error CDF.

The CDF of the localization error is depicted in Figure 4, where it can be observed that the REKF-TQ achieves 90th percentile at a localization error of about 1.8 m, whereas the errors of R-IMM, REKF and EKF are 2.1 m, 2.2 m and 2.6 m, respectively. Most of the localization errors of REKF-TQ are lower than R-IMM, REKF and EKF, meaning the proposed algorithm has a better performance and higher localization accuracy.

**Figure 5:**
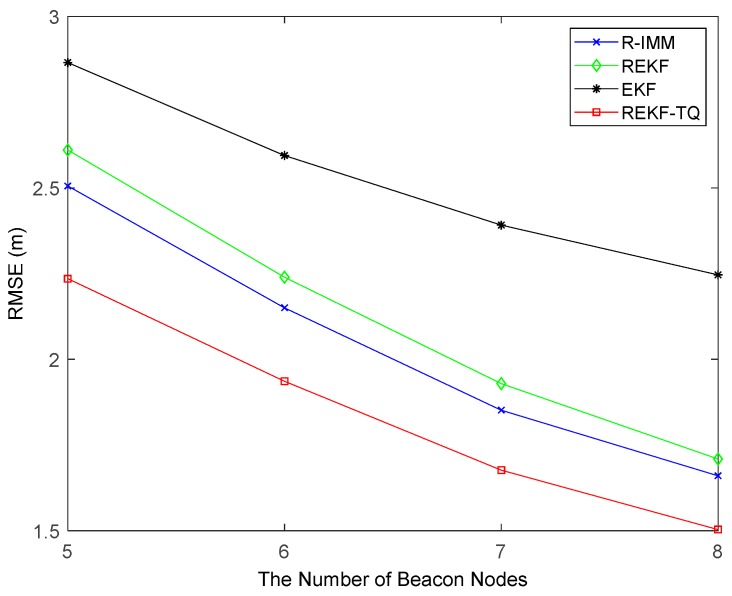
The RMSE versus the number of beacon nodes.

Figure 5 provides the RMSEs of the trackers with the number of beacon nodes varying from 5 to 8. Evidently, the proposed method also has the best positioning precision compared with other methods. And the REKF-TQ has higher localization accuracy in terms of average RMSE than R-IMM, REKF, and EKF, about 9.98%, 13.42% and 27.22%, respectively.

**Figure 6:**
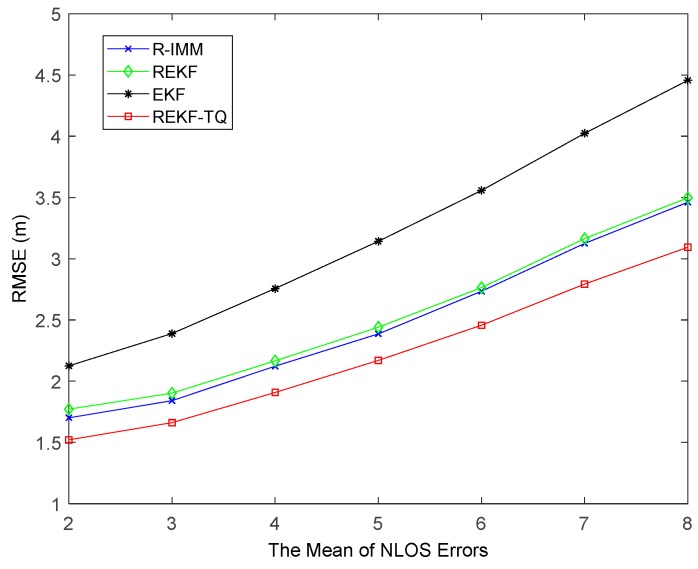
The RMSE versus mean of NLOS errors.

In order to further verify the effectiveness and robustness of REKF-TQ, the paper investigates the behavior of the proposed algorithm at changing the mean of NLOS error, the standard deviation of NLOS error and the probability of LOS propagation. Figure 6 shows that with mean of NLOS errors varying from 2 to 8, the positioning errors of all methods increase. As expected the REKF-TQ achieves best performance and has improved according to the average RMSE relative to R-IMM, REKF, and EKF, by 11.33%, 11.88%, and 43.87%, respectively.

**Figure 7:**
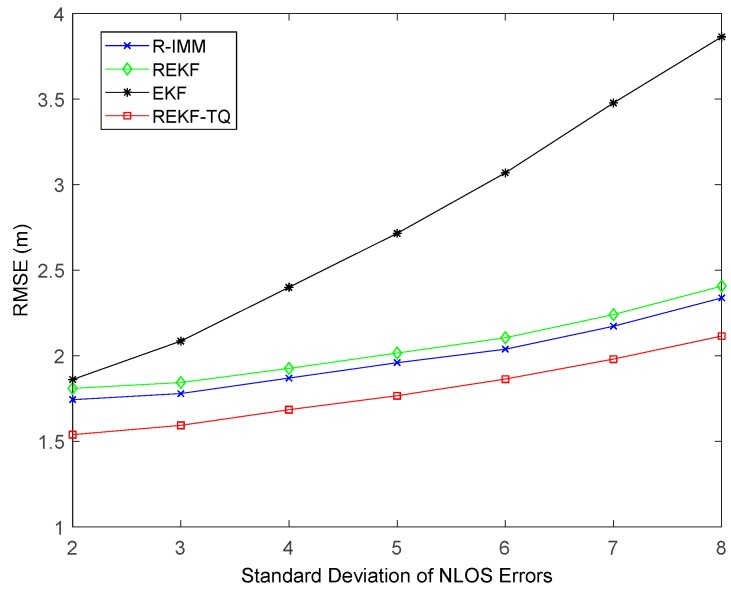
The RMSE versus standard deviation of NLOS errors.

Figure 7 displays the effect of the standard deviation of NLOS errors on these methods. It is obvious to observe that at all points the proposed algorithm achieves the best performance. The average RMSE values of REKF-TQ, R-IMM, REKF and EKF are 1.79 m, 1.98 m, 2.05 m and 2.78 m, respectively.

**Figure 8:**
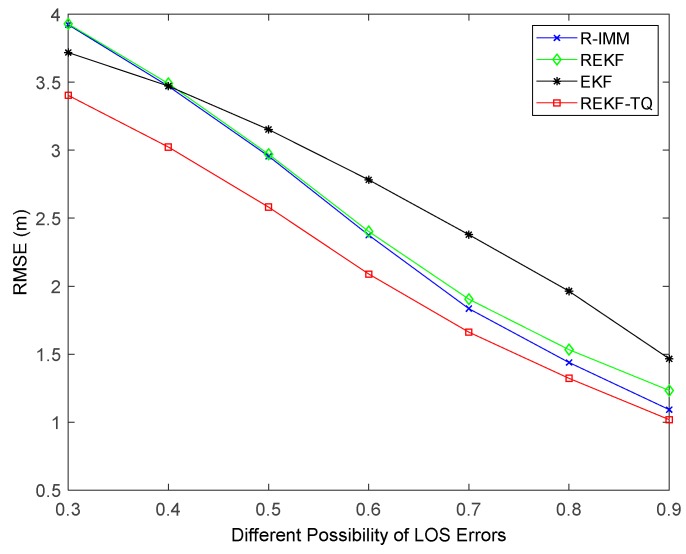
The RMSE versus the probability of LOS propagation.

Simulation results of changing the probability of LOS propagation are illustrated in Figure 8. It can be observed that when the LOS probability is small, the EKF performs better than the R-IMM and REKF. That is because any bounded score function has a breakdown point when more than 50% of the measurements are acquired from the nodes in NLOS environment [33]. By contrast, the REKF-TQ achieves highest accuracy in the case of both small and big probability value, which demonstrates the feasibility and validity of the proposed algorithm.

#### 5.1.3. The NLOS Errors Obey Exponential Distribution

The default parameters obeying an exponential distribution are listed in Table 2.

**Figure 9:**
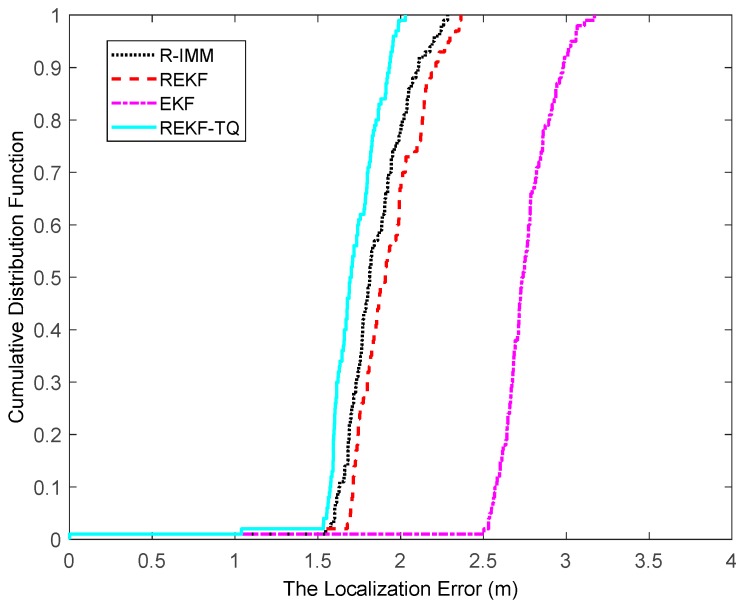
The localization error CDF.

Figure 9 shows the CDF of localization error of different algorithms for the case where the NLOS errors obey an exponential distribution. It can be observed that the ninety-five percent of the REKF-TQ is less than 2.0 m whereas for the R-IMM, REKF, EKF the 95-percentile increases to 2.2 m, 2.3 m and 3.0 m, respectively, which indicates the efficiency and robustness of the proposed algorithm.

**Figure 10:**
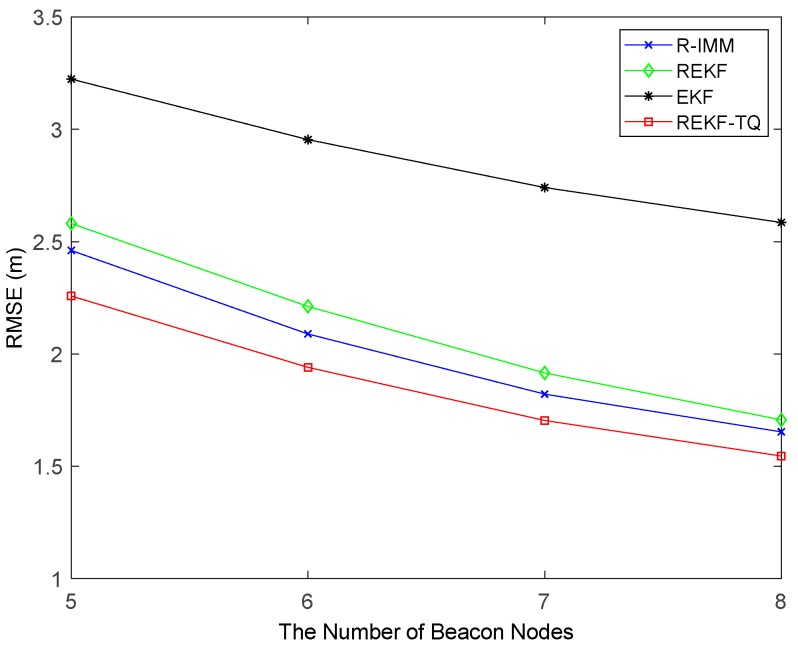
The RMSE versus the number of beacon nodes.

**Figure 11:**
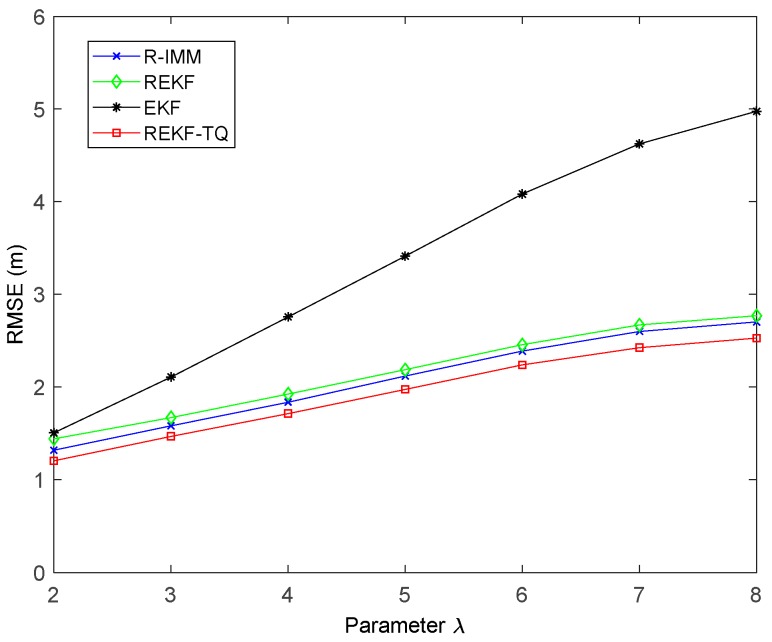
The RMSE versus λ.

**Figure 12:**
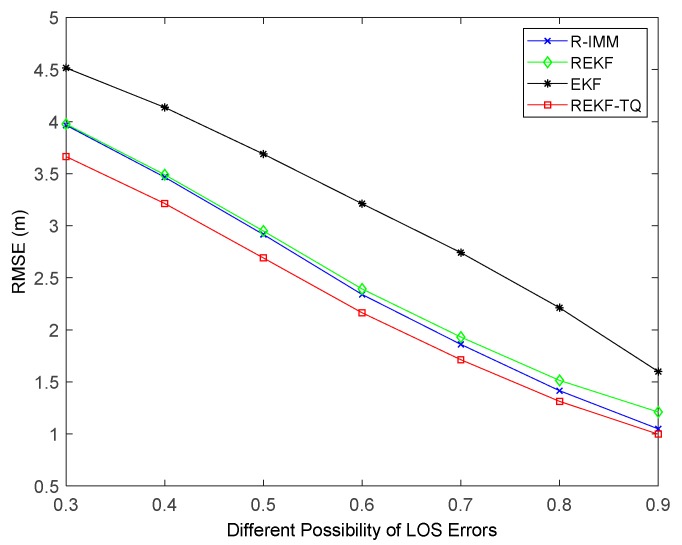
The RMSE versus the probability of LOS propagation.

The impact of the number of beacon nodes on the performance of these algorithm is shown in Figure 10. As the number of nodes increases, all the trackers have smaller RMSE. When six nodes are involved in the positioning process, the REKF-TQ method has higher localization accuracy than R-IMM, REKF, and EKF, about 34.85%, 30.95% and 11.20%, respectively. Figure 11 shows the effect of the parameter λ in the simulation while Figure 12 presents the result of changing the LOS propagation probability. As the value of the parameter λ gets larger in Figure 11, the EKF position error increases sharply, which indicates it achieves poor performance in the situation where large deviation occurs between the measurements and the true values. At the same time, the REKF-TQ displays great robustness in the environment of serious error contamination and acquires the average RMSE error of 1.94 m while 2.08 m for R-IMM, 2.16 m for REKF and 3.35 m for EKF. As the NLOS probability increases gradually, as show in Figure 12, the position errors of all the algorithms become larger and REKF-TQ still obtains the most accurate state estimation. When the LOS probability is 0.6, the RMSEs of R-IMM, REKF, EKF and proposed algorithm are 2.34 m, 2.39 m, 3.21 m and 2.16 m, respectively.

#### 5.1.4. The NLOS Errors Obey Uniform Distribution

The default parameter values in the simulation are shown in Table 3.

**Figure 13:**
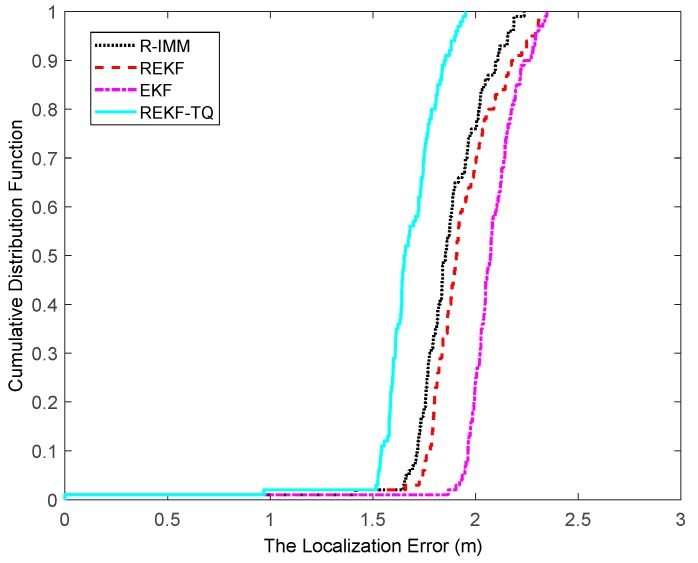
The localization error CDF.

Figure 13 shows the CDF of localization error when the pdf of NLOS error is modeled by a uniform distribution. It can be seen that ninety percent is at approximately 1.8 m for REKF-TQ, at 2.1 m for R-IMM, at 2.2 m for REKF and at 2.3 m for EKF. The proposed algorithm still leads to higher precision in the NLOS condition.

**Figure 14:**
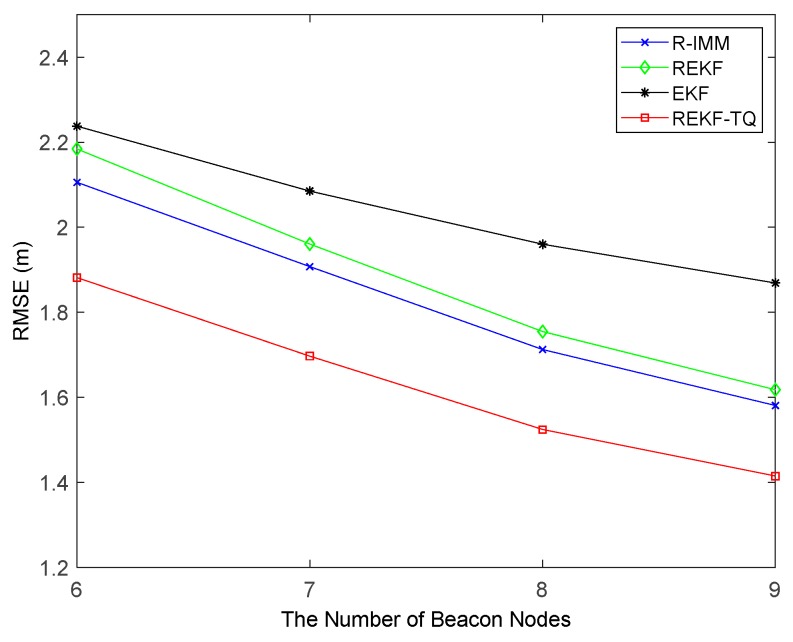
The RMSE versus the number of beacon nodes.

Figure 14 displays the relationship between the number of beacon nodes and the estimation error. The performance of all algorithms has improved to a certain extent when the node number increases. And the proposed method has higher localization accuracy than R-IMM, REKF and EKF, about 10.78%, 13.30% and 20.10%, respectively. Further results are summarized in Table 4.

Changing the model of NLOS error distribution leads to similar localization results whereas the positioning accuracy of the REKF-TQ algorithm rather remains stable compared to other methods. This is because the proposed algorithm combines the advantages of the two filtering methods, i.e., the efficiency of EKF in LOS case and the robustness of REKF when NLOS occurs. Whether in the case of LOS or NLOS, one of these two filters has better performance, and can obtain more accurate positioning results. Through the customized standardized distance function, it can be easy to qualitatively determine which filter has better performance, which will be given a larger weight to ensure the accuracy of the estimation in the final fusion process. Moreover, the proposed algorithm fully exploits the information about the location estimation at last moment to carry out the one of the present moment, making it has self-adaptability and real-time, and can effectively correct the positioning results, so it can be more accurate under different NLOS error distributions. Interacting multiple model (IMM) algorithm, which includes multiple filters, realizes location and tracking through the interaction of REK and REKF. The probability of each model can be adjusted adaptively such that the positioning accuracy is improved compared with EKF and REKF. But the models in IMM algorithm may not match the reality well, so it has some limitations and cannot always obtain a more accurate location estimation.

### 5.2. Experimental Results

#### 5.2.1. Localization Results Analysis

To detect the performance of the algorithm in real environment, two real experiments are designed and carried out in indoor environment. The measurements are obtained by the Ultra Wideband technology (UWB), which is a carrier-free communication technology and uses nanosecond non-sinusoidal narrow pulse signal to transmit data.

**Figure 15:**
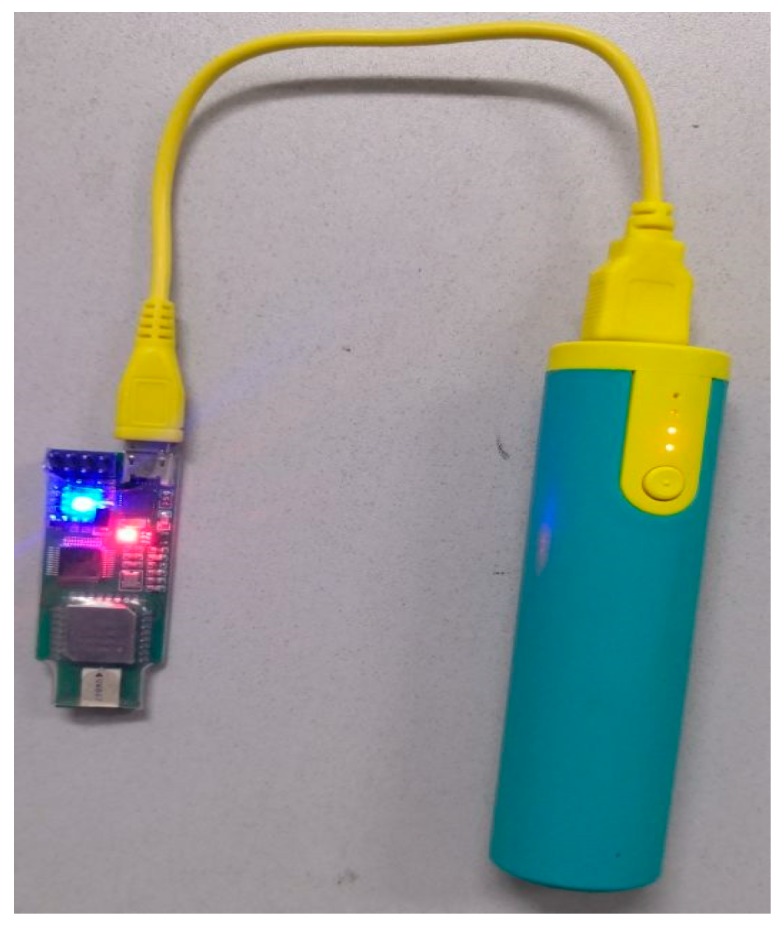
The UWB node.

UWB signals are sent in the form of short and intermittent pulses at very high speed (transmission rate can reach 500 megabits per second) over a very wide spectrum (up to 7.5 megahertz). The UBW system can transmit data with a small power (only several hundred microwatts to tens of milliwatt). The receiver collects signals from the whole signal bandwidth and reconstructs UWB pulses. In indoor positioning, the UWB can accurately measure the flying time of radio signals, and then calculate the distance between the two devices. Combined with geometric positioning methods such as triangular positioning, the position information of the target can be easily obtained. It has been frequently used for accurate indoor positioning in recent years for its characteristics. The UWB device used in the experiments is shown in Figure 15. The ranging time is 15 milliseconds, i.e., one distance data can be obtained every 15 milliseconds. The maximum communication distance is up to 50 m and the communication frequency ranges from 3.5 megahertz to 6.5 megahertz. On the left side of the figure is the UWB node and right is the power supply of the node.

**Figure 16:**
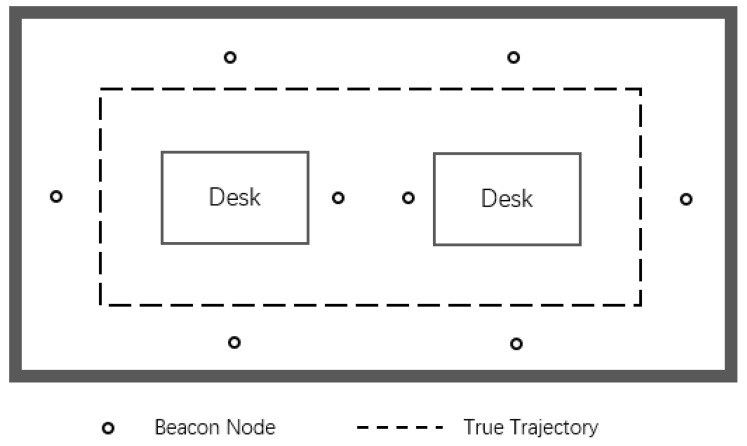
The floor plan of the first experimental environment.

In the first experiment, as shown in Figure 16, eight beacon nodes are placed in a 14 × 6 $m^{2}$ room. The mobile node moves along a rectangular trajectory and travels 36 sampling points at a speed of 60 cm per second, and the corresponding measurement value of each sampling point is used for position estimation.

**Figure 17:**
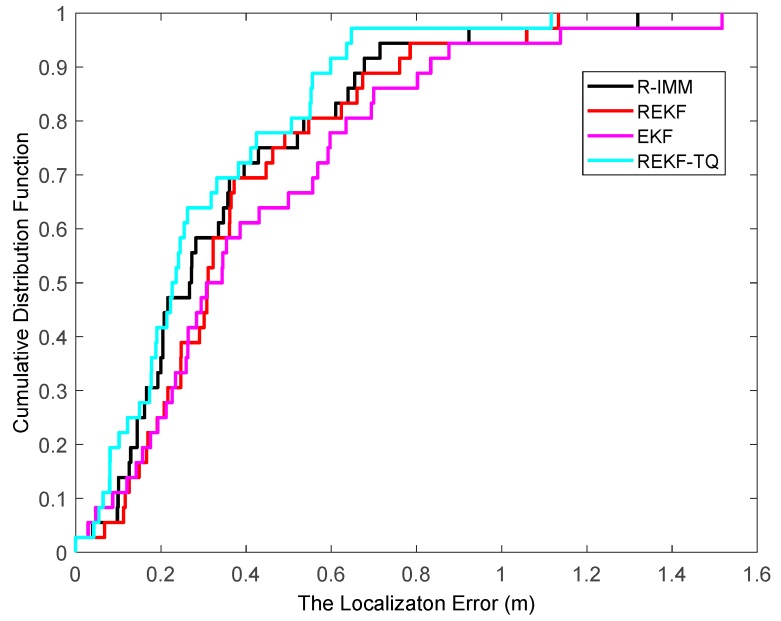
The localization error CDF.

The cumulative distribution function of estimation error in the first experimental condition is shown in Figure 17. The average localization error of the R-IMM, REKF, EKF and REKF-TQ is 0.437 m, 0.456 m, 0.530 m and 0.373 m, respectively. Ninety-five percent of the localization error of the proposed algorithm, R-IMM, REKF and EKF are less than 0.63 m, 0.72 m, 0.78 and 0.88 m. The experiment shows that the proposed algorithm has high positioning accuracy and strong robustness.

**Figure 18:**
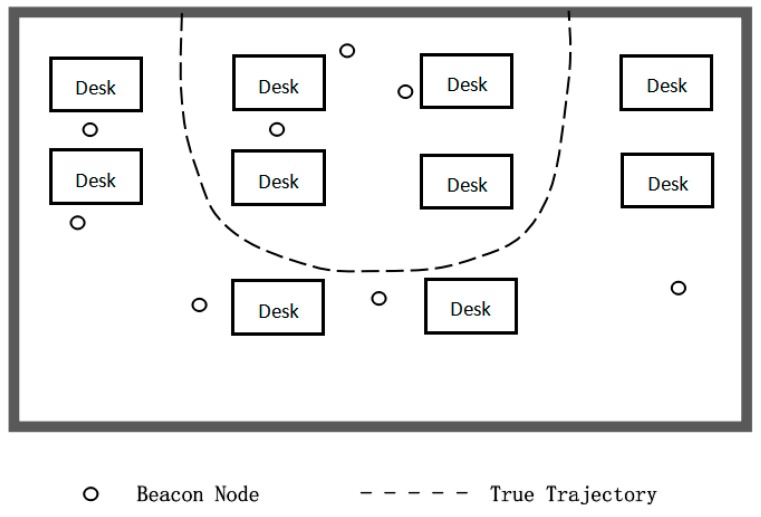
The floor plan of the second experimental environment.

The second experiment is carried out under the condition that beacon nodes are randomly arranged in a 10 × 7 $m^{2}$ room and the mobile node in Figure 18 moves along a curved trajectory, which is similar to the simulation scenario. Sampling one point on the trajectory per second for a total of 31 and the corresponding measurement value of each sampling point is used for position estimation.

**Figure 19:**
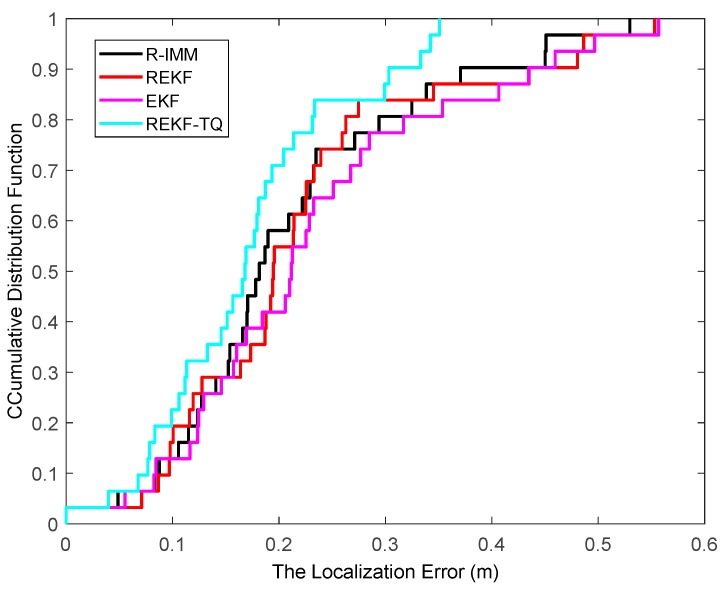
The localization error CDF.

Figure 19 displays the cumulative distribution function of estimation error in the second experimental condition. It can be seen that the ninety-five percent of the REKF-TQ is less than 0.34 m while for the R-IMM, REKF, EKF the 95-percentile is 0.45 m, 0.49 m and 0.50 m, respectively, which indicates the performance of the proposed algorithm is obviously better than any of the other three algorithms even in harsh indoor environment. The results of these two realistic experiments fully illustrate the effectiveness, robustness and practicability of the proposed algorithm.

#### 5.2.2. Computation Time

Table 5 shows the running times of the R-IMM, REKF, EKF and REKF-TQ. All the methods are performed using Matlab2014 and tested on a Windows 10 Professional workstation with Intel(R) Core(TM) i5-6200U CPU @2.30 GHz 2.40GHz and 8.00 GB RAM. Since the REKF is incorporated into the framework of R-IMM and REKF-TQ, their running time is slightly longer than REKF.

## 6. Conclusions

In this paper, an algorithm using a robust extended Kalman filter and a fusion method based on the weighted track quality is proposed to mitigate NLOS errors. It is assumed that only the sensor noise variance is known. The extended Kalman filter (EKF) equations have been reformulated as a linearized regression model, which allows us to apply robust estimation techniques. In particular, this paper considers the Huber’s maximum likelihood-type estimator to robustify the EKF to achieve the robustness in NLOS environment. However, the efficiency in the nominal case and the robustness in the NLOS case are contradictory indicators so that the robust extended Kalman filter (REKF) loses precise positioning performance in the case of LOS. Instead, an EKF with the REKF is concurrently used for preliminary location estimation. The position estimates obtained from the two filters are considered as observation vectors, which can be processed in the framework of Kalman filter (KF). Then two KFs with new observation vector and equation are run in parallel to further filter the estimates using the feedback information based on the location result of the previous moment. Finally, the acquired estimates are combined by the fusion algorithm based on the track quality to get the final position vector of mobile node. Simulation results illustrate that the proposed algorithm generates the accurate location estimation, and it significantly outperforms the EKF, REKF and even the interacting multiple model algorithm (IMM) with EKF and REKF in the LOS case and NLOS environment.

## Figures and Tables

**Table 1:** The default parameters of a Gaussian distribution.

Parameter	Symbol	Default Values
The number of beacon nodes	*N*	7
The probability of LOS propagation	ε	0.7
The standard deviation of measurement noise	$\sigma_{LOS}$	1
The NLOS errors	$N\left(\mu_{NLOS},\sigma_{NLOS}^{2}\right)$	$N\left(3,4^{2}\right)$
The number of sample points	*K*	100
The number of Monte Carlo runs	$T_{n}$	1000

**Table 2:** The default parameters of an exponential distribution.

Parameter	Symbol	Default Values
The number of beacon nodes	*N*	7
The probability of LOS propagation	ε	0.7
The standard deviation of measurement noise	$\sigma_{LOS}$	1
The NLOS errors	E(λ)	E(4)
The number of sample points	*K*	100
The number of Monte Carlo runs	$T_{n}$	1000

**Table 3:** The default parameters of a uniform distribution.

Parameter	Symbol	Default Values
The number of beacon nodes	*N*	7
The probability of LOS propagation	ε	0.7
The standard deviation of measurement noise	$\sigma_{LOS}$	1
The NLOS errors	$U(U_{\min},U_{\max})$	U(0,7)
The number of sample points	*K*	100
The number of Monte Carlo runs	$T_{n}$	1000

**Table 4:** The mean value of error, standard deviation of error, root mean squared error (in m) of estimators for Gaussian, exponential and uniform NLOS Distributions.

	Mean Value of Error	Standard Deviation of Error	Root Mean Squared Error
	Gaussian Distribution $\left(\mu_{NLOS},\sigma_{NLOS}^{2})=(3,4^{2}\right)$		
R-IMM	1.396	0.179	1.887
REKF	1.457	0.184	1.948
EKF	1.988	0.158	2.406
REKF-TQ	1.338	0.143	1.701
	Exponential Distribution λ=4		
R-IMM	1.359	0.180	1.845
REKF	1.436	0.181	1.921
EKF	2.187	0.193	2.760
REKF-TQ	1.348	0.139	1.731
	Uniform Distribution $\left(U_{\min},U_{\max})=(0,7\right)$		
R-IMM	1.459	0.158	1.885
REKF	1.526	0.162	1.954
EKF	1.790	0.116	2.086
REKF-TQ	1.389	0.112	1.685

**Table 5:** Running time of each algorithm.

Method Used	Running Time/s
R-IMM	0.77
REKF	0.64
EKF	0.02
REKF-TQ	0.79
